# Cognitive Styles and Psychotic Experiences in a Community Sample

**DOI:** 10.1371/journal.pone.0080055

**Published:** 2013-11-14

**Authors:** Sarah Sullivan, Richard P. Bentall, Charles Fernyhough, Rebecca M. Pearson, Stanley Zammit

**Affiliations:** 1 Academic Centre for Mental Health Research, University of Bristol, Bristol, United Kingdom; 2 Institute of Psychology, Health and Society, University of Liverpool, Liverpool, United Kingdom; 3 Department of Psychology, Durham University, Durham, United Kingdom; 4 Institute of Psychological Medicine and Clinical Neurosciences, Cardiff University, Cardiff, United Kingdom; Baylor College of Medicine, United States of America

## Abstract

**Introduction:**

In clinical populations paranoid delusions are associated with making global, stable and external attributions for negative events. Paranoia is common in community samples but it is not known whether it is associated with a similar cognitive style. This study investigates the association between cognitive style and paranoia in a large community sample of young adults.

**Methods:**

2694 young adults (mean age 17.8, SD 4.6) from the ALSPAC cohort provided data on psychotic experiences and cognitive style. Psychotic experiences were assessed using a semi-structured interview and cognitive style was assessed using the Cognitive Styles Questionnaire-Short Form (CSQ-SF) on the same occasion. Logistic regression was used to investigate associations between paranoia and CSQ-SF scores, both total and domain-related (global, stable, self, external). The role of concurrent self-reported depressive symptoms in the association was explored.

**Results:**

Paranoia was associated with Total CSQ-SF scores (adjusted OR 1.69 95% CI 1.29, 2.22), as well as global (OR 1.56 95% CI 1.17, 2.08), stable (OR 1.56 95% CI 1.17, 2.08) and self (OR 1.37 95% CI 1.05, 1.79) domains, only Total score and global domain associations remained after additional adjustment for self-reported depression. There was no association between paranoia and external cognitive style (OR 1.10 95% CI 0.83, 1.47).

**Conclusion:**

Paranoid ideation in a community sample is associated with a global rather than an external cognitive style. An external cognitive style may be a characteristic of more severe paranoid beliefs. Further work is required to determine the role of depression in the association between cognitive style and paranoia.

## Introduction

Paranoia, “the tendency to believe that others hold malevolent intentions towards the self” [1] is common in psychosis [2-4]. For example, patients have delusions about being spied on, or persecuted. The common feature of these delusions is the belief that the self is the target of the malevolent intentions of others.

Paranoia is also common in nonclinical populations [5-7], with a prevalence of broadly-defined paranoia of 10%. However, there may be qualitative differences between paranoia in clinical and community populations. Paranoid patients, compared to non-patients, feel less control over their beliefs [8] and their symptoms are less sensitive to context [9]. Also, individuals with paranoia in community samples are much more likely to believe that persecution is deserved because of personal fault (‘bad-me’ paranoia) [10] whereas patients usually have either exclusively ‘poor-me’ paranoia when persecution is believed to be undeserved. They may also fluctuate between poor-me and bad-me beliefs [11,12].

Paranoia is associated with distress and help-seeking in both clinical [13] and general population samples [14] and therefore attempts have been made to understand it in terms of cognitive processes [15,16]. A focus on the individual’s cognitive style (i.e. making attributions or causal explanations about salient events), may be useful. The Attributional Theory of paranoia was developed following the observation that paranoid patients made excessively external (blaming others or the circumstances), stable (unchangeable) and global (assuming that the cause will affect all areas of life) attributions for hypothetical negative events [17]. Bentall et al [18] explains this in terms of latent negative self-schemata that are easily activated when faced with a threat to a preferred view of the self. In order to avoid negative self-thoughts, an external attribution is made to reduce the potential discrepancy between the ideal and actual self. The disadvantage of this process is that it may activate beliefs that others view the individual negatively. It is argued that the recurrent use of this kind of reasoning may result in elaborate suspicions about others and hence paranoia. 

The Attributional Model has therapeutic implications because attributional style is modifiable by cognitive therapy and related interventions [19,20]. However evidence in its favour is at best mixed. Early studies found that paranoia was associated with an excessive self-serving bias (i.e. a bias towards attributing positive events to internal causes and negative events to external causes) which is greater than that found in healthy people [21-23], a finding that is consistent with the model. However, subsequent studies have failed to replicate these findings [24]. There are several possible explanations for this inconsistency. First, a variety of assessment tools have been used to measure attributional style, which make different assumptions about the way in which it should be measured (for example, about whether it is important to distinguish between external attributions that implicate others and those that implicate situational factors) [25] [26] [27]. Second, studies have been conducted in both clinical and general populations, which may differ in the quantitative and qualitative features of paranoia. Two studies [28] [29] found an abnormal self-serving bias in clinical but not community samples and another [11] found an abnormal attributional style in poor-me but not bad-me patients. Another study, with a student sample, only found an association between paranoia and an excessive self-serving bias in a sub-group who scored very highly for paranoia [30]. Third, much of the existing evidence has been hampered by the lack of adjustment for co-morbid symptoms such as depression. Depression is common in paranoid patients and known to influence attributional style [31], but is rarely controlled for. Hallucinations are a frequently observed co-morbid symptom in clinical populations [32], although at present there are no compelling theoretical reasons to assume that they are associated with attributional bias. No study to date has investigated the relationship between hallucinations and cognitive style. A final limitation of existing studies is sampling. The majority have used clinical and/or convenience samples rather than a representative community sample. This is important given evidence that paranoia in clinical and general population samples may be qualitatively different.

This study investigates the association between attributional style and paranoia and hallucinations in a large community sample of young adults, using a measure of attributional style [33]. Hallucinations were the most prevalent experience and rarely occurred alongside any of the other experiences. This allowed us to investigate the specificity of attributional style for paranoia by investigating its association with hallucinations, and by controlling for co-morbid depression. 

We hypothesise that paranoid beliefs will be associated with an excessive tendency to make external, stable and global attributions for negative events. There are no specific predictions for hallucinations.

## Materials and Methods

### The Avon Longitudinal Study of Parents and Children (ALSPAC)

 ALSPAC recruited 14,541 pregnant women resident in Avon, UK with expected dates of delivery 1^st^ April 1991 to 31^st^ December 1992. Of these initial pregnancies, there was a total of 14, 676 foetuses, resulting in 14, 062 live births and 13,988 children who were alive at 1 year of age. When the oldest children were approximately 7 years of age an additional 713 children were enrolled resulting in a total sample size of 15,458 foetuses. Of this total sample 14,701 were alive at 1 year. The cohort has been previously described[34,35] and is representative of the UK population [36].

### Ethics Statement

Ethical approval for the study was obtained from the ALSPAC Law and Ethics Committee and the Southmead, Frenchay, UBHT and Weston Research Ethics Committees. Written consent was obtained from participants to allow use of anonymized linked data for research by bona fide scientists.

### Measures: Outcome

#### Psychotic experiences age 18

The Psychosis-Like Symptom interview (PLIKSi) [37] is a semi-structured instrument that uses the principles of standardised clinical examination developed for the Schedule for Clinical Assessment in Psychiatry (SCAN). It consists of 11 ‘core’ questions eliciting key psychotic experiences occurring since age 12, covering hallucinations (visual and auditory), delusions (being spied on, persecution, thoughts being read, reference, control, and grandiosity), and experiences of thought interference (broadcasting, insertion and withdrawal). Any unspecified delusions elicited were also rated. Cross-questioning was used to establish the presence of psychotic experiences, and coding of items followed the glossary definitions and rating rules for SCAN. Interviewers were psychology graduates trained in assessment using the SCAN Psychosis Section and in the PLIKSi. Interviewers rated experiences as not present, suspected, or definitely present. Unclear responses after probing were always ‘rated down’, and experiences only rated as definite when a credible example was provided. Interviewers discussed cases with a psychiatrist if it was unclear how an experience should be rated. At regular intervals interviewer ratings for a sample of recorded interviews were confirmed by a psychiatrist to ensure interviewers were rating experiences correctly. If the interviewer rated the experience as suspected or definitely psychotic they were asked whether experiences reported were always attributable to the effects of sleep (hypnopompic or hypnogogic experiences), fever, or substance use (defined as experiences occurring only within 2 hours of intoxication with drugs or alcohol). If the experiences were attributable to these causes they were not used in this analysis.

For the purposes of this analysis the outcome variables used were total psychotic experiences, hallucinations only, and persecutory delusions only. Each was used as a binary variable (no experiences versus suspected or definite experiences).

#### Reliability

Test-retest agreement for any psychotic experience was 0.73 (SE=0.074), and was higher for interviews rated by the same interviewer at both time points (kappa=0.86, S.E=0.136). For a detailed description see Zammit et al 2013[38].

### Exposure

#### Attributional Style at age 18

A short form of the Cognitive Style Questionnaire (CSQ-SF) [33], developed from the longer Cognitive Style Questionnaire [39], was administered to the ALSPAC children at a clinic. The reliability and validity of the short version has been established in young adults [33], although the reliability of one domain (internal) is lower than the others. The measure focuses on 8 negative hypothetical events relating to failures in academic, employment and interpersonal relationships. For each event participants are asked to vividly imagine themselves in that situation and think carefully about the likely reason for the event. Participants then rate the extent to which this reason was caused by internal versus external factors (caused by themselves or others), specific versus global factors (will impact all areas of life or just this specific situation), stable versus unstable factors (the cause will persist and lead to the same outcome in the future) and reflects their self-worth (they are flawed in some way) on Likert scales of agreement from 1-5. For each scenario, two items related to each of 4 dimensions resulting in 8 items across 8 scenarios, 64 items in total. When calculating total scores, we summed the domains so that high total scores indicated high scores for stability, globality, implications for self and *externality* (rather than internality), because this was the pattern anticipated for paranoia based on previous research [40]. In order to derive an externality score the internality domain score was reversed. This way of totalling the scores is different from that used in depression research, where the internality dimension is scored so that a high score implies internal attributions. The range of the total score was from 64 to 320 and the domain scores from 16 to 80, with higher scores indicating a style hypothesised to be associated with psychotic experiences. Total score and domain scores were computed and showed normal distributions. Internal consistency for the total score in a comparable ALSPAC sample was α= 0.89 and for the domain scores was; stability α=0.74, self α=0.84, globality α=0.69 and externality α=0.56 [41] which is comparable to previous studies [33]. A principal-components analysis of the scores for the four dimensions indicated that a single factor with an eigenvalue of 3.25 explained 65% of the variance [41]. Three of the domains (globality, stability and self-worth) were highly correlated with each other (range r=0.56-0.63) although the externality domain was less strongly correlated (range r=0.19-0.32) [41]. These findings are consistent with findings from other attributional measures [33,42]. The measure was administered as an interviewer-assisted questionnaire, with respondents verbally providing the answer to the stem question of each scenario.

### Potential Confounders

Potential confounders were selected using clinical knowledge of variables associated with community psychotic experiences and which have been used in previous research on cognitive style. The child-specific variables selected were: gender, IQ at age 15 measured by alternate items of the Weschler Abbreviated Scale of Intelligence (WASI) and self-reported depression measured using the Short Moods and Feelings Questionnaire (SMFQ)[43] at age 18. The family-specific variables selected were: maternal education and marital status at the child’s birth, home ownership status and social class (highest of mother’s and father’s) also measured at the child’s birth.

A median split of the CSQ-SF total score resulted in two groups: high and low. The distribution of each potential confounder was examined across the median split. Those with higher total CSQ-SF scores were more likely to be female with higher self-reported depression scores at 18 years and to have a mother of higher social class (see Table 1). These items were selected as potential confounding variables using the following rationale. Gender is likely to be related to psychotic experiences [44] and may also be associated with cognitive style [45]. There is existing evidence that social class is associated with the probability of psychotic experiences [46]. Finally, self-reported depression is associated both with cognitive style [41] and with psychotic experiences [47].

**Table 1:** Demographic characteristics of total sample (n=2694) and across high and low categories of total CSQ scores split at the median.

Variable	Level/Measure	Total sample	High CSQ-sf total scores mean (SD)	Low CSQ-sf total scores mean (SD)
Gender %	M	41.2%	36.9%	45.6%
	F	58.8%	63.05%	54.4%
IQ at 15 mean (SD)		94.7 (12.6)	94.9 (13.1)	94.6 (12.1)
Self-reported depression at 18 years mean (SD)	SMFQ range (0-26)	6.4 (5.2)	7.6 (5.4)	5.2 (4.6)
Psychotic experiences at 12 years %	suspected or definite psychotic experiences	12.7%	13.4%	12.1%
Ethnicity %	White	95.6%	95.0%	96.1%
Maternal educational status %	Low (CSE, vocational, O level)	49.4%	53.0%	51.8%
	High (A levels, degree) %	50.6%	47.0%	48.7%
Maternal marital status %	Never married	11.8%	12.8%	11.0%
	Married presently or previously	88.2%	87.3%	89.1%
Social class (highest of maternal and paternal) %	I & II	35.1%	37.0%	33.2%
	III manual and non-manual	50.3%	47.6%	52.9%
	IV & V	14.6%	15.4%	13.9%
Home ownership status %	Mortgaged/owned	87.4%	87.2%	87.5%
	Rented	12.6%	12.8%	12.5%

Abbreviations: SMFQ – Short Moods and Feelings Questionnaire

Dataset

**Figure 1:**
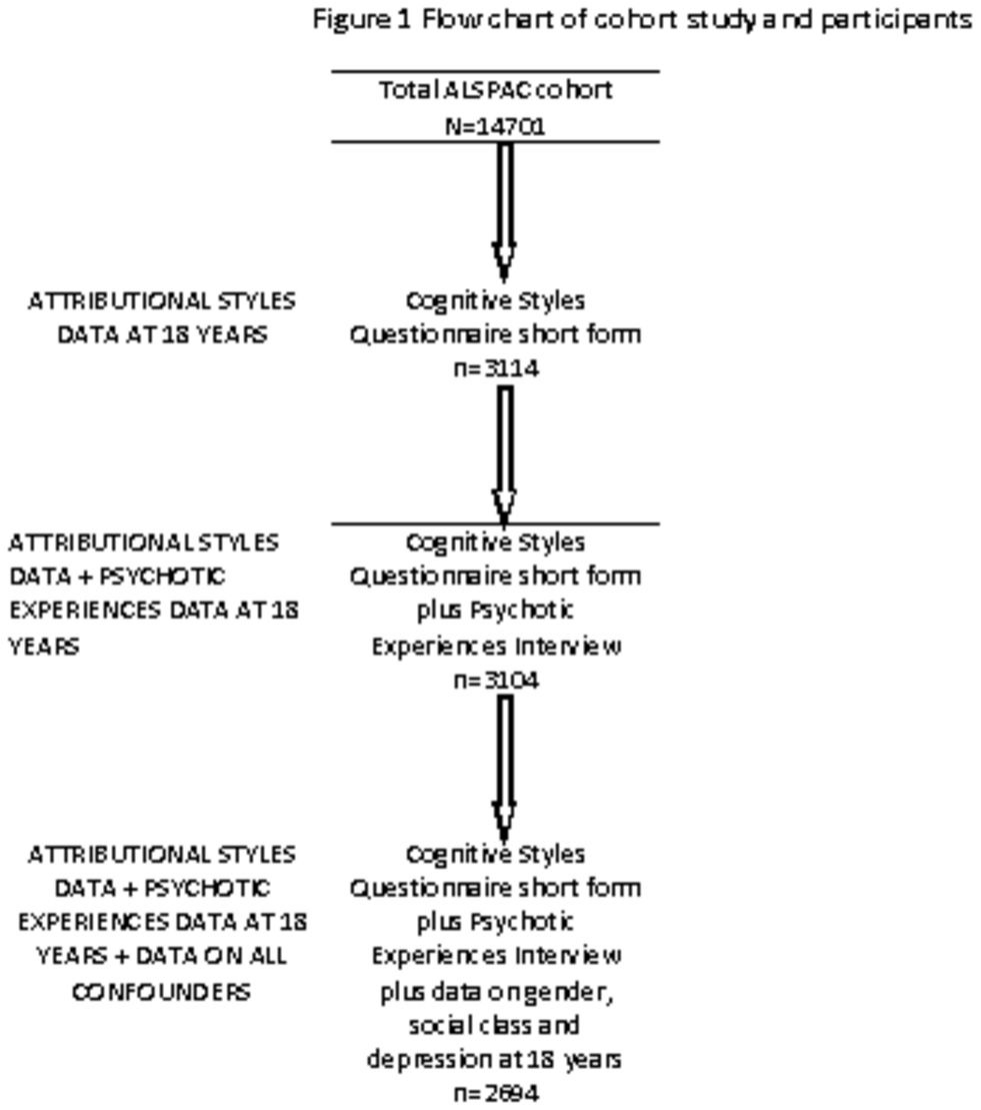
Flow chart of cohort and study participants.

The dataset consists of a group of the cohort for whom data were available on the outcome, exposure and all confounders (n=2694) (see Figure 1).

### Statistical Analysis

Logistic regression models were used to investigate associations between total CSQ-SF score, all CSQ-SF score domains and the presence of suspected or definite psychotic experiences. Confounding variables, including concurrent self-reported depression, were used to adjust the logistic regression models. 

Although there were missing data previous studies on the ALSPAC cohort using imputed data have shown that the results are not materially different and therefore bias resulting from loss to follow-up has not affected the findings[48]. Consequently a complete cases analysis (i.e. all cases with data on the outcome, exposure and all confounding variables) was conducted.

 Two sensitivity analyses were also performed. The first investigated whether any association was influenced differentially by those with extremely high or low CSQ-SF scores. In order to investigate this possibility the analyses above were repeated but excluded those with CSQ-SF total (number excluded = 17) and CSQ-SF domain scores (number excluded stable n=8, self n=22, global n=17 and external n=13) which were 3 SD or more above or below the mean. It is possible that early psychotic experiences may result in an altered cognitive style at 18 years, therefore the second sensitivity analysis investigated the influence of early psychotic experiences by repeating the analysis excluding participants who had also experienced suspected or definite psychotic experiences at age 12 (n=309).

## Results

 The participants included in this study were more likely to have parents in social classes I and II and to be female than the rest of the cohort. They were also less likely to have had psychotic experiences including paranoia (see Table 2).

**Table 2:** Descriptive data comparing the ALSPAC cohort (n=15602) and study dataset (n=2694).

Variable	Time point (years)	Range/categories	Cohort		Dataset	
			Proportion	Mean (SD)	Proportion	Mean (SD)
Total psychotic experiences	18	Suspected and definite/no	9.13%		8.61%	
Hallucinations	18		8.76%		8.28%	
Paranoia	18		2.36%		1.89%	
Cognitive style questionnaire - total score	18	64-320		161.97 (20.03)		162.05 (20.07)
Cognitive style questionnaire - stable score	18	16-80		39.81 (6.68)		39.85 (6.62)
Cognitive style questionnaire - self score	18	16-80		35.91 (8.11)		35.93 (8.11)
Cognitive style questionnaire - global score	18	16-80		38.53 (6.19)		38.44 (6.21)
Cognitive style questionnaire - external score	18	16-80		55.27 (5.99)		55.18 (6.00)
Gender	Birth	M	51.36%		41.26%	
Parental social class	Birth	Classes I and II	26.05%		35.09%	
Depressive symptoms score	18	0-26		6.55 (5.23)		6.44 (5.16)

Suspected or definite psychotic experiences at age 18 were detected in 232 (8.6%) participants; 20 of the 232 (8.6%) had experienced paranoia alone, 191 of the 232 (82.3%) had hallucinations alone and 21 of the 232 (9.1%) both paranoia and hallucinations. There was evidence of a statistical association between hallucinations and paranoia (χ ^2^ =189.64 p<0.0001) and evidence of a cross-sectional association between self-reported depressive symptoms (SMFQ scores) at 18 years and psychotic experiences (OR 1.12 95% CI 1.10, 1.15), paranoia (OR 1.12 95% CI 1.08, 1.17) and hallucinations (OR 1.12 95% CI 1.10, 1.15). 

Those who had higher CSQ-SF scores were more likely to be female, have more depressive symptoms and psychotic experiences and to have parents of a lower social class (see Table 1).

The unadjusted analysis showed strong evidence of an association between paranoid delusions and CSQ-SF total score, and with all CSQ-SF sub-domains excepting the external CSQ-SF domain (see Table 3). The associations were attenuated by adjusting for gender and social class. Adjustment for concurrent self-reported depression further attenuated these associations, although there was still evidence of association between both CSQ-SF total scores and global scores and paranoid delusions.

**Table 3:** Odds ratios, 95% confidence intervals and p values of the association between CSQ-sf scores and psychotic experiences (y/n).

	Unadjusted paranoia	Adjusted 1	Adjusted 2	Unadjusted hallucinations	Adjusted 1	Adjusted 2
CSQ-sf total (SD=18)	1.75 (1.34, 2.27) ≤0.0001	1.69 (1.29, 2.22) ≤0.0001	1.34 (1.01, 1.78) 0.04	1.28 (1.11, 1.46) ≤0.0001	1.27 (1.11, 1.47) 0.001	1.03 (0.89, 1.19) 0.71
CSQ-sf stable (SD=7)	1.57 (1.19, 2.09) 0.002	1.56 (1.17, 2.08) 0.003	1.25 (0.93, 1.68) 0.30	1.17 (1.01, 1.35) 0.03	1.19 (1.03, 1.38) 0.02	0.97 (0.83, 1.13) 0.66
CSQ-sf self (SD=7)	1.43 (1.11, 1.85) 0.005	1.37 (1.05, 1.79) 0.02	1.11 (0.86, 1.46) 0.41	1.09 (0.95, 1.24) 0.23	1.09 (0.95, 1.26) 0.20	0.90 (0.78, 1.04) 0.15
CSQ-sf global (SD=6)	1.64 (1.28, 2.09) ≤0.0001	1.66 (1.30, 2.14) ≤0.0001	1.36 (1.05, 1.77) 0.02	1.30 (1.14, 1.48) 0.0001	1.30 (1.14, 1.49) ≤0.0001	1.07 (0.93, 1.23) 0.34
CSQ-sf external (SD=6)	1.18 (0.89, 1.56) 0.26	1.10 (0.83, 1.47) 0.51	1.16 (0.87, 1.55) 0.22	1.19 (1.04, 1.37) 0.01	1.15 (1.00, 1.33) 0.05	1.21 (1.05, 1.40) 0.01

Adjusted 1 – gender and social class (highest of mother’s and father’s)

Adjusted 2 – gender and social class (highest of mother’s and father’s)+ self reported depression at 18

Key – CSQ-sf Cognitive Styles Questionnaire – short form

Results expressed in terms of SD increase in CSQ-sf scores. Complete cases n=2694

Hallucinations were associated with CSQ-SF total scores and with the stable, global and external CSQ-SF domains in the unadjusted analysis. These remained after adjustment for gender and social class, but after further adjustment for concurrent depressive symptoms only the association with the external domain remained. 

When the outcomes of paranoid delusions and hallucinations were directly compared there was evidence that the association between paranoia and CSQ-SF scores is stronger than the association between hallucinations and CSQ-SF scores for total CSQ-SF and all domains of the CSQ-SF, except for the external domain (see Table S1).

The association between paranoia and the CSQ-SF total and global domain scores may have been driven by those at the extreme end of the CSQ-SF score distribution. When the analyses above were repeated excluding any participants who scored more than three SDs above or below the mean, the associations between experience of paranoia and the CSQ-SF total score and the CSQ-SF global score were attenuated: OR 1.25 95% CI 0.92, 1.70 and OR 1.23 95% CI 0.91, 1.67 respectively. However the association between experience of hallucinations and CSQ-SF external domain score remained even after removing those with extreme scores; OR 1.26 95% CI 1.08, 1.46 (see Table S2).

Repeating the analyses excluding those who had experienced psychotic experiences at 12 years (n=309), the associations between paranoia and total and global domain CSQ-SF scores were attenuated (OR 1.22 95% CI 0.93, 1.73 and OR 1.17 95% CI 0.81 and 1.68) (see Table S3). 

## Discussion

### Main findings

Our hypothesis that paranoid delusions would be associated with higher global, stable and external CSQ-SF domain scores was only partially supported. Although we found associations between CSQ-SF total and global scores and paranoia, we did not find an association with external or stable CSQ-SF domain scores. Moreover, unexpectedly, hallucinations were associated with the external CSQ-SF domain scores.

The findings for paranoia are not in accordance with earlier studies in clinical samples [21,22,40]. However they are in accordance with more recent studies with community samples. One study [28] found an association between an externalising bias and paranoia in a clinical group but not in a community sample at high risk of psychosis (n=88). Another, using student participants (n=114) [49], found no evidence of an association between attributional bias and paranoia across the whole sample, however in a subgroup with less depression, moderate anxiety and high levels of self-esteem, (a group which it was suggested may be a poor-me sub-type), there was evidence of a personalising bias (a tendency to excessively attribute negative events to external-personal causes). A third study [30] found only found evidence of an association between an externalising style and paranoia in a sub-group with severe paranoia. 

### Differences between the Present Study Findings and Previous Studies

This is the first large study of cognitive style in relation to paranoia using a representative community sample. 

Previous studies have not adopted a consistent approach to minimising confounding, particularly for mood. A failure to adjust for depression may under-estimate the association between CSQ externalising scores and paranoia and may over-estimate associations between paranoia and global, stable and self CSQ domains. In our analyses, the associations between externalising and both hallucinations and paranoia were increased by adjusting for depression. 

### Hallucinations

Unexpected associations were observed between CSQ-SF scores and hallucinations in this study. Although both paranoia and hallucinations load on the same positive factor in analyses of patients’ symptoms [32], the relationship between the two types of experiences is probably complex. Some have argued that delusions and perceptual abnormalities result from the same cognitive deficits [50]. Others have pointed to evidence that delusional beliefs can influence source monitoring abnormalities thought to underlie hallucinations [51] but also that anomalous experiences can provoke delusional thinking [52]. A recent study of first-episode patients reported that delusions and hallucinations began within the same month in 45% of cases, delusions preceded hallucinations in 19% of cases, hallucinations preceded delusions in 16% of cases and 20% developed delusions in the absence of hallucinations [53]. In the ALSPAC sample the proportion of those experiencing both hallucinations and delusions at 18 years of age was surprisingly low. Together, these findings suggest that the association between hallucinations and delusions may be different at different points in the evolution of psychosis.

Contrary to prediction, the presence of hallucinations, rather than paranoia, was associated with an excessively external attributional style for negative events. One possibility is that the experience of hallucinations leads individuals to suppose that negative events are caused by external forces.

### Limitations

 The CSQ-SF only measures attributions for hypothetical negative events, in contrast to other measures which assess causal explanations for positive and negative events. Additionally the external domain of the CSQ-SF has relatively poor psychometric qualities and may have been affected by measurement error which would reduce the probability of detecting an association.

In the study dataset only 1.3% (n=35) had a psychotic disorder. These people would be more likely to have an extreme attributional style and may be a reason why an association was not found with some CSQ-SF domains.

At present it is not known what proportion of young people with psychotic experiences at 18 years will develop a psychotic illness and it is likely that many will only experience sub-syndromal psychotic states during their lifetimes. 

Although designed to reduce measurement error our measure of psychotic experiences was used as a binary variable and therefore does not reflect important factors such as frequency and severity.

There was a marked reduction in the strength of the relationships between CSQ-SF scores and paranoia at 18 years when those who had experienced paranoia or hallucinations at 12 years were excluded. One possible explanation is that psychotic experiences in early adolescence cause an abnormal cognitive style or that an abnormal cognitive style in childhood is more strongly associated with early adolescent psychotic experiences than with those occurring in early adulthood. Unfortunately we do not have earlier measures of cognitive style to enable us to examine either of these possibilities. It is also possible that the attenuated associations are due to the reduction of statistical power when the sample was reduced.

The cross-sectional design of our study does not allow us to investigate whether cognitive style causes paranoia or vice versa.

### Implications

 Several possible implications may be drawn from our findings. Firstly, the paranoid experiences of clinical and community populations may be qualitatively different. In our community sample paranoia was associated with a more pessimistic cognitive style i.e. that the causes of the negative event would impact on many other areas. By contrast, in clinical groups paranoia appears to be associated not only with a global and stable cognitive style but also one that attributes the cause of negative findings to others. This represents a cognitive style which many would consider to be more characteristic of paranoid behaviour. Secondly, it is possible that early on in its developmental trajectory paranoia is associated with a global cognitive style which later becomes more stable and external. Thirdly, it is possible that a stable, external and global style is associated with more severe or frequent episodes of paranoia which may be under-represented in the study dataset. The final possibility concerns the lack of certainty about the meaning of community psychotic experiences. Several recent findings in the ALSPAC cohort suggest that they may be an expression of psychological distress rather than an endophenotype of psychosis. There is some evidence (unpublished data) that a global style is also associated with depressive symptoms (another form of psychological distress) in and that psychotic experiences and depressive symptoms are strongly correlated (further work conducted by the authors, details available on request) suggesting an underlying common psychological construct. 

This is a clinically relevant finding as psychological interventions for people with the psychotic symptom of paranoia typically target attributional and other reasoning processes. One of the attributional styles most actively targeted is externality. Our findings suggest that it is also key to target a global style which may be important in the development of paranoia and therefore also in reducing paranoid symptomology. Interestingly, one study found that patients treated with CBT for paranoid beliefs sometimes benefited without showing a shift in attributional style [54]. 

Future studies addressing the psychology of paranoid thinking need to consider subtypes of paranoia (mild vs severe; poor-me vs bad-me) and the developmental evolution of paranoid thinking (subclinical vs prodromal vs first episode vs chronic). Longitudinal studies with repeated measures are required to investigate the development of cognitive style and its relationship with the emergence of depression and psychotic experiences over time.

## Supporting Information

Table S1 **Odds ratios & 95% confidence intervals for multinomial logistic regression of: no experiences-reference group (0), hallucinations only (1), paranoia only (2), both (3) (may have other experiences too).** Complete cases n=2693.
(DOCX)

Table S2 **Odds ratios and 95% confidence intervals of the association between CSQ-sf scores and psychotic symptom (y/n); in a sample without extreme CSQ scores (i.e. >3SDs from mean)-CSQ total 19 excluded, CSQ stable 9 excluded, CSQ self 26 excluded, CSQ global 20 excluded, CSQ internal 14 excluded.**
(DOCX)

Table S3 **Odds ratios and 95% confidence intervals of the association between CSQ-sf scores and psychotic symptoms (y/n).** Results expressed in terms of SD increase in CSQ-sf scores. Complete cases omitting those with PE suspected or definite at age 12 n=2385.
(DOCX)
