# Supplementary material for: Cognitive Styles and Psychotic Experiences in a Community Sample
Source: PLoS One. 2013 Nov 14;8(11):e80055. doi: 10.1371/journal.pone.0080055 (PMC3828222; doi:10.1371/journal.pone.0080055)
Supplement: Table S1 — Odds ratios & 95% confidence intervals for multinomial logistic regression of: no experiences-reference group (0), hallucinations only (1), paranoia only (2), both (3) (may have other experiences too). Complete cases n=2693. (DOCX) [file pone.0080055.s001.docx]

Table S1

Odds ratios & 95% confidence intervals for multinomial logistic regression of: no experiences-reference group (0), hallucinations only (1), paranoia only (2), both (3) (may have other experiences too). Complete cases n=2693

|  | Reference group – no experiences y/n | Unadjusted Hallucinations y/n | Adjust 1 | Adjust 2 | Unadjusted Paranoia y/n | Adjust 1 | Adjust 2 | Unadjusted Hallucinations & paranoia y/n | Adjust 1 | Adjust 2 |
| --- | --- | --- | --- | --- | --- | --- | --- | --- | --- | --- |
| CSQ-sf total (SD=18) | 1.00 | 1.24 (1.07, 1.44) | 0.99 (0.86, 1.15) | 1.01 (0.87, 1.18) | 2.13 (1.43, 3.15) | 1.77 (1.16, 2.69) | 1.77 (1.15, 2.73) | 1.58 (1.12, 2.22) | 1.19 (0.85, 1.66) | 1.25 (0.87, 1.77) |
| CSQ-sf stable (SD=7) | 1.00 | 1.15 (0.98, 1.34) | 0.94 (0.80, 1.09) | 0.95 (0.81, 1.12) | 2.00 (1.30, 3.07) | 1.63 (1.05, 2.54) | 1.69 (1.07, 2.67) | 1.36 (0.95, 1.97) | 1.04 (0.73, 1.48) | 1.10 (0.76, 1.60) |
| CSQ-sf self (SD=7) | 1.00 | 1.06 (0.92, 1.22) | 0.86 (0.75, 0.99) | 0.88 (0.76, 1.02) | 1.67 (1.14, 2.45) | 1.42 (0.96, 2.09) | 1.40 (0.93, 2.10) | 1.30 (0.93, 1.81) | 0.95 (0.70, 1.31) | 1.06 (0.76, 1.49) |
| CSQ-sf global (SD=6) | 1.00 | 1.28 (1.11, 1.47) | 1.04 (0.90, 1.20) | 1.06 (0.91, 1.23) | 1.93 (1.35, 2.78) | 1.63 (1.11, 2.38) | 1.64 (1.10, 2.44) | 1.51 (1.10, 2.09) | 1.23 (0.90, 1.68) | 1.23 (0.87, 1.72) |
| CSQ-sf external (SD=6) | 1.00 | 1.19 (1.02, 1.38) | 1.23 (1.07, 1.42) | 1.22 (1.05, 1.42) | 1.10 (0.71, 1.71) | 1.09 (0.71, 1.66) | 1.12 (0.72, 1.75) | 1.25 (0.87, 1.80) | 1.38 (0.99, 1.94) | 1.30 (0.90, 1.87) |

Adjusted 1 – gender and maternal educational status

Adjusted 2 – gender and maternal educational status + self reported depression at 18
