# Supplementary material for: Cognitive Styles and Psychotic Experiences in a Community Sample
Source: PLoS One. 2013 Nov 14;8(11):e80055. doi: 10.1371/journal.pone.0080055 (PMC3828222; doi:10.1371/journal.pone.0080055)
Supplement: Table S2 — Odds ratios and 95% confidence intervals of the association between CSQ-sf scores and psychotic symptom (y/n); in a sample without extreme CSQ scores (i.e. >3SDs from mean)-CSQ total 19 excluded, CSQ stable 9 excluded, CSQ self 26 excluded, CSQ global 20 excluded, CSQ internal 14 excluded. (DOCX) [file pone.0080055.s002.docx]

Table S2

Odds ratios and 95% confidence intervals of the association between CSQ-sf scores and psychotic symptom (y/n); in a sample without extreme CSQ scores (i.e. >3SDs from mean)-CSQ total 19 excluded, CSQ stable 9 excluded, CSQ self 26 excluded, CSQ global 20 excluded, CSQ internal 14 excluded

|  | Unadjusted paranoia | Adjusted 1 | Adjusted 2 | Unadjusted hallucinations | Adjusted 1 | Adjusted 2 |
| --- | --- | --- | --- | --- | --- | --- |
| CSQ total | 1.59 (1.19, 2.12) | 1.50 (1.11, 2.02) | 1.25 (0.92, 1.70) | 1.23 (1.07, 1.42) | 1.26 (1.09, 1.45) | 1.02 (0.88, 1.19) |
| CSQ stable | 1.39 (1.02, 1.88) | 1.42 (1.05, 1.93) | 1.19 (0.87, 1.63) | 1.12 (0.96, 1.29) | 1.14 (0.98, 1.32) | 0.93 (0.80, 1.09) |
| CSQ self | 1.18 (0.88, 1.59) | 1.20 (0.89, 1.62) | 1.03 (0.76, 1.39) | 0.98 (0.85, 1.13) | 0.99 (0.86, 1.15) | 0.83 (0.71, 0.96) |
| CSQ global | 1.44 (1.08, 1.91) | 1.43 (1.07, 1.91) | 1.23 (0.91, 1.67) | 1.31 (1.14, 1.50) | 1.30 (1.13, 1.50) | 1.09 (0.94, 1.27) |
| CSQ external | 1.17 (0.88, 1.56) | 1.14 (0.85, 1.52) | 1.20 (0.90, 1.61) | 1.21 (1.05, 1.40) | 1.19 (1.02, 1.37) | 1.26 (1.08, 1.46) |

Adjusted 1 – gender and maternal educational status

Adjusted 2 – gender and maternal educational status + self reported depression at 18
