# Supplementary material for: Cognitive Styles and Psychotic Experiences in a Community Sample
Source: PLoS One. 2013 Nov 14;8(11):e80055. doi: 10.1371/journal.pone.0080055 (PMC3828222; doi:10.1371/journal.pone.0080055)
Supplement: Table S3 — Odds ratios and 95% confidence intervals of the association between CSQ-sf scores and psychotic symptoms (y/n). Results expressed in terms of SD increase in CSQ-sf scores. Complete cases omitting those with PE suspected or definite at age 12 n=2385. (DOCX) [file pone.0080055.s003.docx]

Table S3

Odds ratios and 95% confidence intervals of the association between CSQ-sf scores and psychotic symptoms (y/n). Results expressed in terms of SD increase in CSQ-sf scores. Complete cases omitting those with PE suspected or definite at age 12 n=2385

|  | Unadjusted paranoia | Adjusted 1 | Adjusted 2 | Unadjusted hallucinations | Adjusted 1 | Adjusted 2 |
| --- | --- | --- | --- | --- | --- | --- |
| CSQ total (SD=18) | 1.43 (0.99, 2.06) | 1.45 (1.00, 2.10) | 1.22 (0.83, 1.78) | 1.30 (1.10, 1.53) | 1.31 (1.11, 1.55) | 1.08 (0.91, 1.28) |
| CSQ stable (SD=7) | 1.32 (0.90, 1.93) | 1.35 (0.92, 1.99) | 1.13 (0.76, 1.69) | 1.21 (1.02, 1.43) | 1.24 (1.04, 1.57) | 1.01 (0.84, 1.21) |
| CSQ self (SD=7) | 1.09 (0.76, 1.56) | 1.12 (0.78, 1.61) | 0.95 (0.66, 1.37) | 1.09 (0.93, 1.27) | 1.10 (0.94, 1.30) | 0.92 (0.78, 1.08) |
| CSQ global (SD=6) | 1.36 (0.96, 1.91) | 1.37 (0.97, 1.95) | 1.17 (0.81, 1.68) | 1.30 (1.11, 1.51) | 1.30 (1.11, 1.52) | 1.08 (0.92, 1.28) |
| CSQ external (SD=6) | 1.40 (0.96, 2.05) | 1.39 (0.94, 2.03) | 1.45 (0.99, 2.14) | 1.22 (1.03, 1.44) | 1.20 (1.01, 1.42) | 1.28 (1.07, 1.51) |

Adjusted 1 – gender and maternal educational status

Adjusted 2 – gender and maternal educational status + self reported depression at 18
